# Purification, Characterization and *in vitro* Evaluation of Polymyxin A From *Paenibacillus dendritiformis*: An Underexplored Member of the Polymyxin Family

**DOI:** 10.3389/fmicb.2018.02864

**Published:** 2018-11-23

**Authors:** Manoj Jangra, Harmandeep Kaur Randhawa, Manpreet Kaur, Anugya Srivastava, Navdezda Maurya, Prashant P. Patil, Pallavi Jaswal, Ashish Arora, Prabhu B. Patil, Manoj Raje, Hemraj Nandanwar

**Affiliations:** ^1^Clinical Microbiology and Bioactive Screening Laboratory, CSIR-Institute of Microbial Technology, Chandigarh, India; ^2^Bacterial Genetics and Genomics Laboratory, CSIR-Institute of Microbial Technology, Chandigarh, India; ^3^Cell Biology and Microscopy Laboratory, CSIR-Institute of Microbial Technology, Chandigarh, India; ^4^Molecular and Structural Biology Division, CSIR-Central Drug Research Institute, Lucknow, India

**Keywords:** polymyxin A, gram-negative infections, *Paenibacillus dendritiformis*, antibiotic-resistance, antimicrobial agent

## Abstract

Nosocomial infections caused by antibiotic-resistant Gram-negative pathogens are of grave concern today. Polymyxins are considered as the last resorts of therapy to treat these multi-drug resistant (MDR) bacteria. But their associated nephrotoxicity and neurotoxicity calls for the development of safer polymyxin therapy until novel and less toxic antibiotics are discovered. No other polymyxin molecule except polymyxin B and E (colistin) is explored thoroughly in literature to demonstrate its clinical relevance. In the present study, we have isolated two antimicrobial compounds named P1 and P2 from the soil isolate *Paenibacillus dendritiformis* strain PV3-16, which we later identified as polymyxin A_2_ and A_1_ respectively. We tested their minimum inhibitory concentrations (MICs) against MDR clinical isolates, performed membrane permeabilization assays and determined their interaction with lipopolysaccharide (LPS). Finally, we studied their toxicity against human Leukemic monocyte cell line (THP-1) and embryonic kidney cell line (HEK 293). Both compounds displayed equal efficacy when compared with standard polymyxins. P1 was 2–4 fold more active in most of the clinical strains tested. Moreover, P1 showed higher affinity toward LPS. In cytotoxicity studies, P1 had IC_50_ value (>1000 μg/ml) similar to colistin against HEK cells but immune cells, i.e., THP-1 cell lines were more sensitive to polymyxins. P1 showed less toxicity in THP-1 cell line than all other polymyxins checked. To sum up, P1 (polymyxin A_2_) possessed better efficacy than polymyxin B and E and had least toxicity to immune cells. Since polymyxin A was not investigated thoroughly, we performed the comprehensive *in vitro* assessment of this molecule. Moreover, this is the first report of isolation and characterization of polymyxin A from *P. dendritiformis*. This compound should be further investigated for its *in vivo* efficacy and toxicity to develop it as a drug candidate.

## Introduction

The emergence of Multi-Drug Resistant (MDR), Extensive Drug-Resistant (XDR), and Pan Drug-Resistant (PDR) bacterial strains is an alarming threat to our society. This is in part because of the fact that our modern drug and discovery development has faltered (Payne et al., 2007; Kollef et al., 2011; Magiorakos et al., 2012). *Escherichia coli* and four other Gram-negative microorganisms in the ‘ESKAPE’ pathogens category, also called Gram-negative Bacilli (GNB), are responsible for the major nosocomial and community infections worldwide (Tzouvelekis et al., 2012; Vasoo et al., 2015). These superbugs have acquired resistance to most widely used beta-lactams, cephalosporins, and even the last line drugs such as carbapenems (Boucher et al., 2009; Livermore, 2009; Marchaim et al., 2012; Hu et al., 2016; Rhouma et al., 2016). Many times polymyxins, which reappeared in the early 2000s, remain the last options to treat deadly infections caused by Carbapenem-resistant *Enterobacteriaceae*, MDR *Acinetobacter baumannii*, and *Pseudomonas aeruginosa*. No antibiotic with less toxicity and better potential than polymyxin B and colistin is available to fight multidrug-resistant gram-negative bacteria hitherto (Gelband et al., 2015).

Polymyxins are cyclic cationic polypeptide antibiotics, discovered in the 1940s (Storm et al., 1977). They consist of decapeptide attached to a fatty acid chain at its N-terminus. The five main polymyxins (A to E) were initially described in literature along with other polymyxins such as M, S, and T (Shoji et al., 1977a,b; Storm et al., 1977; Martin et al., 2003). Among them, only polymyxin B and E (colistin) were studied extensively and used in the clinical setting. Polymyxin B and E differ just at one amino acid position (D-Phe at position 6 in polymyxin B whereas D-Leu in polymyxin E). Due to the high structural similarity, there is no significant difference between their toxicity and biological activity (Oliveira et al., 2009; Gales et al., 2011). Nephrotoxicity is the most observed and studied adverse effect associated with these polymyxins. In old literature, neurotoxicity has also been a concern but it is not as understood as nephrotoxicity. Also, neurotoxicity cases of polymyxins are low (0–5% patients) (Falagas and Kasiakou, 2006; Landman et al., 2008; Justo and Bosso, 2015; Pogue et al., 2017). Comparative toxicity has not been evaluated in details in literature. Roberts et al. (2015) observed that main lipopeptides in polymyxin B displayed higher toxicity than colistin under *in vitro* conditions, but there was no significant difference when studied *in vivo* using mice models. However, according to one recent study, patients receiving polymyxin B therapy showed less renal toxicity as compared to those receiving colistin (Rigatto et al., 2016). Other reports also suggested the potential advantage of polymyxin B in comparison to colistin regarding the toxicity problem (Akajagbor et al., 2013; Phe et al., 2014). The mechanism for this reduced toxicity of polymyxin B is unknown, but in some cases, still, colistin is preferred over polymyxins B since it is available as methanesulfonate pro-drug (Couet et al., 2011; Sandri et al., 2013). As polymyxins serve as sole weapons until novel and effective antibiotics are discovered, there is still need for a safer polymyxin therapy to treat MDR Gram-negative infections (Landman et al., 2008).

During the screening for new antimicrobial agents, we isolated and purified two molecules from a bacterial strain belonging to *Paenibacillus* genus. After chemical and structural characterization, we proposed them as polymyxin A_1_ and A_2_. In the literature, polymyxin A has been reported very scarcely. We determined their minimum inhibitory concentration (MIC) against various MDR clinical isolates and examined their toxicity in two mammalian cell lines. We also studied their effects on the membrane of Gram-negative bacteria via different techniques. Additionally, we analyzed their biosynthetic gene cluster using whole genome sequencing. To the best of our knowledge, here we report for the first time the isolation and characterization of polymyxin A from *P. dendritiformis* strain PV3-16. We have performed comprehensive *in vitro* assessment of polymyxin A and its comparison with polymyxin B and E.

## Methodology

### Isolation of Bacteria and Antimicrobial Screening

We isolated the microbes from two different niches: soil and water samples from Leh and Ladakh, India. The samples, after serial dilution in normal saline, were spread plated on three different media, i.e., R2A agar (HiMedia), tryptic soy agar (HiMedia) and Actinomycete isolation agar (HiMedia) and incubated for 1 week at 30 and 37°C. All purified isolates were preserved in 20% glycerol stock at -80°C. Isolates were grown in tryptic soy broth for 24–96 h and crude extracts were prepared using Diaion HP-20 resin (Sigma) as explained in Section “Purification of Antimicrobial Compound(s) From PV3-16 Strain.” Antimicrobial activity of cell-free supernatants and extracts was assessed using agar well diffusion assay (Valgas et al., 2007) with the test strain seeded in the molten agar. Test strains used were: *E. coli* ATCC 25922, *Klebsiella pneumoniae* ATCC 29665, *S. aureus* ATCC 25923 and *C. albicans* ATCC 10231. Positive isolates were identified based on 16S rRNA gene sequencing. The isolate PV3-16, previously unreported for antimicrobial production, was selected for further study.

### Purification of Antimicrobial Compound(s) From PV3-16 Strain

Two to three colonies from the freshly grown plate were inoculated into tryptic soy broth and incubated at 37°C overnight. This inoculum (1.5%) was transferred to 2 L Erlenmeyer flasks containing 700 ml of sterile R2A broth supplemented with 2% NaCl and incubated till early stationary phase (24–30 h). Cells were removed using centrifugation and cell-free supernatant was allowed to bind to activated Diaion HP-20 (Sigma) resin for 2–3 h. The resin was washed with Milli-Q water and compounds were eluted with a combination of methanol:isopropanol: acetone (60:30:10). 0.01% acetic acid was used to facilitate the elution. The solvent was evaporated under vacuum using rotary evaporation (RotaVap, Heidolph) and reconstituted in Milli-Q water. The crude extract was partially purified using cation-exchange chromatography (SP-Sepharose, GE Healthcare, with 10 mM potassium phosphate buffer, pH 6.5). A linear gradient of NaCl (0–100%) in the same buffer was performed for elution of bound components. Active fractions were pooled and further processed using reversed-phase high-pressure liquid chromatography (RP-HPLC) (C18, XBridge, Waters, 5 μm, 130 Å, 10 × 250 mm). The gradient elution, i.e., 18–35% solvent B (acetonitrile plus 0.075% TFA) over 27 min; 35–95% B in 16 min and reverse 95 to 18% B in 7 min was used in HPLC. Water containing 0.075% TFA was used as solvent A. Flow rate was kept at 3.0 ml/min. All peaks were collected and assayed for bioactivity. The active fractions containing compounds P1 and P2 (RT 16 and 20 min respectively, Supplementary Figure S1) were purified by a second round of HPLC using similar conditions and pure compounds were lyophilized (after acetonitrile removal by rotary evaporation) to get the white powder. The purity of both compounds was assessed by analytical HPLC (Shimadzu UFLC, with LC20AD pump and PDA detector).

### Mass Spectrometry and Amino Acid Analysis

Pure compounds were subjected to MALDI-MS and LC-ESI-MS analysis. LC-ESI-MS data was obtained on TOF/Q-TOF Mass Spectrometer (Agilent Technologies, Model- G6550A). Spectra were recorded in positive-ion mode with spectral range 400–4000 m/z. α-Cyano-4-hydroxycinnamic acid was used as the matrix for sample preparation for MALDI-MS. Mass spectra were recorded on MALDI-TOF mass spectrometer (AB Sciex, 5800 MALDI-TOF/TOF). Tandem MS (MS/MS) analysis was achieved by increasing the LASER intensity and changing other instrumental parameters on the same spectrometer with MALDI-TOF/TOF analyzer. For amino acid analysis, PICO-TAG amino analysis system (Waters) was employed as per operator’s manual. In brief, 100 μg of compound was hydrolyzed at 110°C for 24 h in fumes of 6 N HCl with 1% phenol in a sealed, evacuated tube flushed with nitrogen. Afterward, the solvent was evaporated using vacuum centrifugation (speed-vac) and dried acid-hydrolysate was resuspended in 10–20 μl of ethanol: water: triethylamine mixture (2:2:1) and redried. After that, derivatization was performed by adding the 20 μl derivatizing mixture, i.e., ethanol: triethylamine: water: phenyl isothiocyanate (PITC) in a ratio of 7:1:1:1:1. The tubes were kept at room temperature for 20 min and thoroughly dried under vacuum. Samples were dissolved in 200 μl of 20 mM disodium hydrogen phosphate buffer and analysis was done on HPLC system (Waters, 515 binary Pumps with PDA detector 2996). PICO-TAG column (3.9 × 150 mm, Waters) was used. All standard amino acid mixture provided by manufacturer were derivatized in similar way and processed. Retention time of standard amino acid derivatives was compared with sample hydrolysate. Polymyxin B was used as a positive control and processed in similar conditions.

### NMR Spectrometry

For NMR spectroscopy, the method employed by Martin et al. (2003) for mattacin (polymyxin M) was performed. In brief, both compounds were dissolved at a concentration of 3 mg/ml in 90:10 (H_2_O:D_2_O) solvent. NMR spectra were acquired at 27°C on a Bruker 600 MHz spectrometer coupled with 1.7 mm cryo-probe. One-dimensional ^1^H, ^13^C, and two-dimensional spectra were obtained for both compounds to solve the two-dimensional (2D) structure and the spectra were compared with those of polymyxin M.

### Stereochemical Analysis

Marfey’s analysis [Marfey’s reagent, L-1-fluoro-2-4-dinitrophenyl-5-L-alanine amide (FDAA), Thermo scientific, Catalog number: 48895] was performed to determine the stereochemistry of the Dab residue present at the third position. One milligram of compound P2 was hydrolyzed to its amino acid constituents using the same method as described above (see Mass Spectrometry and Amino Acid Analysis). The hydrolysate was dissolved in Milli-Q (Millipore) and derivation was done as per manufacturer’s instructions. For detection of FDAA derivatives of amino acids, reversed-phase HPLC (XBridge, Waters, C18, 5 μm, 130 Å, 4.6 × 250 mm) was used with mobile phase system: solvent A, water containing 0.075% TFA and solvent B, acetonitrile containing 0.075% TFA. A linear gradient from 20% B to 70% B in 45 min was employed. L-Dab and D, L-Dab from Sigma were used as standard and were derivatized in the same conditions.

### Whole Genome Sequencing and Biosynthetic Gene Cluster Identification

The strain PV3-16 was grown in TSB medium overnight, and genomic DNA was isolated using ZR Fungal bacterial DNA Miniprep kit (Zymo research) as per the manufacturer’s instructions. For whole genome sequencing, Illumina *de novo* sequencer was used. In brief, Illumina sequencing libraries were prepared using Nextera XT with dual indexing adaptors from Illumina. Tagmentation time was kept 5 min, and targeted size for cleanup was <500 bp. Illumina libraries were sequenced on Illumina Miseq using 2 × 150 bp paired-end run. Illumina reads were *de novo* assembled into the draft genome using CLC Genomics Workbench 7.5. Genome annotation and secondary metabolites cluster analysis were done using RAST (version 2.0) (Aziz et al., 2008) and antiSMASH (version 3.0) (Weber et al., 2015) respectively. NCBI blast was performed for comparative analysis of gene cluster.

### Determination of Minimum Inhibitory Concentration (MIC)

The pathogens used for MIC determination, i.e., *Acinetobacter baumannii* ATCC 19606, *Enterococcus faecalis* ATCC 29212, *Enterococcus faecalis* ATCC 51299, *Escherichia coli* ATCC 35218, *Pseudomonas aeruginosa* ATCC 27853, *Klebsiella pneumoniae* ATCC 700603, *K. pneumoniae* ATCC BAA-1706, *K. pneumoniae* ATCC BAA-2146, *Staphylococcus aureus* ATCC 25923 were procured from HiMedia laboratory, India; *E. coli* ATCC 25922, *K. pneumoniae* ATCC 29665, *Vibrio cholerae* MTCC 3906, *V. parahemolyticus* ATCC 17802, *Bacillus subtilis* ATCC 6633 were obtained from MTCC, Chandigarh, India. Clinical isolates were acquired from Govt. Medical College and Hospital, Chandigarh and Medicose Center, Chandigarh. These clinical strains were isolated from urine, sputum, blood and lung of the patients within the age group of 10–70 years. Antibiotic-susceptibility using agar disk-diffusion method was performed for all the clinical isolates. MIC for both compounds was determined via broth micro-dilution method in cation-adjusted Mueller-Hinton broth (Ca-MHB) using standard Clinical and Laboratory Standards Institute (CLSI) guidelines. 100 μl of Ca-MHB was added in 96-well polystyrene microtiter plate and both compounds along with polymyxin B and E were serially diluted twofold from 32–0.125 μg/ml. Finally, 100 μl of culture containing 10ˆ5 CFU/ml was added and plates were incubated at 37°C for 20–24 h. The MIC was defined as the lowest concentration with no visible growth.

### Time-Kill Kinetics

Log-phase grown cultures of *E. coli* ATCC 25922 and *P. aeruginosa* ATCC 27853 were inoculated into 10 ml fresh MHB in conical flasks to give the final concentration as 10ˆ5 CFU/ml in each flask. For each compound P1, P2 and as control, polymyxin B, the concentration was kept at 2xMIC. The flasks were incubated at 37°C and 200 rpm. At 0, 0.5, 2, 4, 6, 8, and 12 h, 100 μl of appropriately diluted culture was spread plated on MHA plates in triplicates and incubated at 37°C for 24 h. Three plates were used for plating at each time interval and colonies were counted as CFU/ml. One flask was kept as negative control which contained no antibiotic. Two independent experiments were performed on different days.

### Scanning Electron Microscopy

*Klebsiella pneumoniae* BAA-1706, *A. baumannii* ATCC 19606 and *P. aeruginosa* ATCC 27853 were cultured in Ca-MHB medium to mid-exponential phase and harvested at 1500 × *g* for 10 min. The cells were washed with PBS twice and resuspended in PBS to obtain a final OD_600_ of 0.3. The cell suspension was incubated with P1 and P2 (10 μg/ml) at 37°C. The treatment time was 45 min for *K. pneumoniae* and *A. baumanii* while *P. aeruginosa* was incubated for 2 h. After incubation, the cells were washed twice with PBS and fixed in 2.5% (v/v) glutaraldehyde overnight at 4°C. Afterward, the cells were washed with PBS three times and dehydration was performed using graded ethanol steps (30, 50, 70, 90, and 100%), for 30 min each. Thereafter, the bacteria were incubated with tertiary butyl alcohol at room temperature and then at -20°C, for 30 min each. The samples were lyophilized and kept in the desiccator until processed. The cells were gold coated and the specimens were observed under scanning electron microscope (EVO 40XVP CARL ZEISS).

### Membrane Permeabilization Assays

For outer membrane permeabilization, *N*-phenyl-1-naphthylamine (NPN) assay was used as described previously (Qian et al., 2012). *E. coli* ATCC 25922 cells were grown in MHB medium till log phase and cells were washed with 5 mM HEPES buffer (containing 5 mM glucose, pH ∼7.4) twice. Final cell concentration was kept at 2 × 10ˆ6 CFU/ml. NPN was dissolved in acetone at a concentration of 40 mM and then diluted in HEPES buffer to make 40 μM working stock. Two ml of cells were treated with P1, P2 and polymyxin B at 10 μg/ml and 20 μg/ml for 1 h. Afterward, 500 μl of NPN (40 μM) was added to 1500 μl of cells to give final NPN concentration of 10 μM and fluorescence was measured on fluorescence spectrometer with excitation wavelength and emission wavelength, 350 and 420 nm respectively. Cells plus antibiotic without NPN was taken as control for subtracting the background fluorescence. The experiment was performed in triplicates and results were plotted as mean ± SD. For inner membrane permeabilization assay, LIVE/DEAD^®^ BacLight Bacterial Viability Kit (L7012, Molecular Probes, purchased from Thermo Scientific, India) was used and the experiment was performed as per manufacturer’s guidelines. Briefly, log-phase grown cells of *E. coli* ATCC 25922 were washed in normal saline (0.85%) three times and concentration was set to 2 × 10ˆ6 CFU/ml. Cells were treated with 10 μg/ml concentration of both compounds along with polymyxin B as a positive control for 2 h. Two dyes SYTO9 and propidium iodide (PI) were used to stain the cells (as per the manufacture’s protocol) and incubated in dark for 15 min. Fluorescence was measured on fluorescence spectrophotometer with excitation wavelength for both dyes at 485 nm while emission wavelength for SYTO9 was set at 630 nm and for PI, at 530 nm. Cells without antibiotic were taken as negative control. The ratio of SYTO9/PI fluorescence for control cells was taken as 100 percent lives cells. The experiment was done in triplicate.

### Isothermal Calorimetry

LPS (*E. coli* strain 055:B5 origin), polymyxin B and polymyxin E (colistin) were purchased from Sigma-Aldrich. LPS solution and antibiotics were prepared as described previously (Martin et al., 2003). For isothermal titration calorimetry (ITC), titrations were performed on Malvern, auto-iTC 200 system. ITC experiments were carried out at 20°C with reference power at 5 μCal/s. 400 μl of LPS solution (0.05 mM) was loaded in one well while 120 μl of ligands, i.e., P1, P2, polymyxin B and polymyxin E was used at a concentration of 1.0 mg/ml (0.83–0.87 mM). Nineteen injections were given at an interval of 180 s with injection volume 2 μl (injection time was 4 s per injection). The buffer solution was also titrated against LPS to check for the internal interference. Data were analyzed and plotted in Origin 7.0 software suite. Binding isotherms were fit to determine the dissociation constant (Kd) for each compound.

### Mammalian Cytotoxicity

3-(4,5-dimethylthiazol-2-yl)-2,5-diphenyltetrazolium bromide (MTT) assay was employed to examine the cytotoxicity against human leukemic monocyte cell line (THP-1 ATCC TIB-202) and human embryonic kidney cell line (HEK293 ATCC CRL-1573) as explained by Kaur et al. (2017). Toxicity of both compounds was compared with polymyxin B and colistin at concentrations ranging from 62.5 to1000 μg/ml.

## Results

### Identification and Structural Characterization of Antimicrobial Compound(s)

**FIGURE 1:**
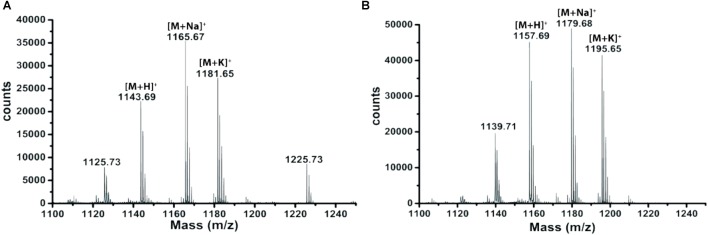
MALDI-MS spectra of P1 **(A)** and P2 **(B).**

**FIGURE 2:**
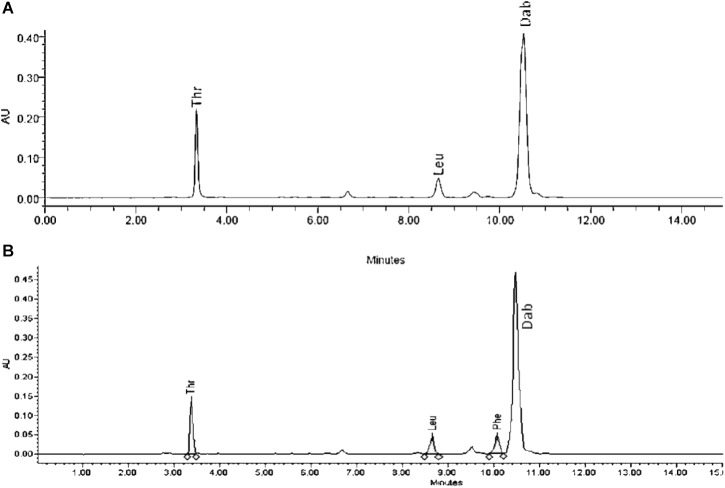
Amino acid analysis of P2 **(A)** and polymyxin B **(B).**

**FIGURE 3:**
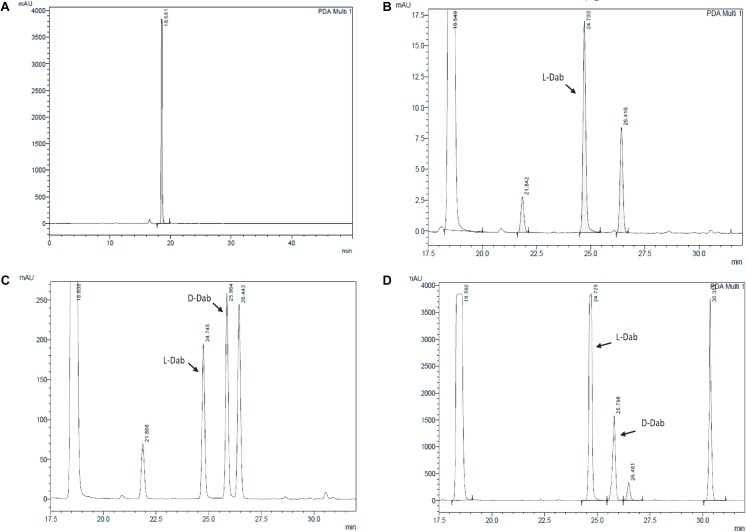
Stereochemical analysis of P2 using Marfey’s reagent. **(A)** Control Marfey’s reagent **(B)** Standard L-Dab **(C)** Standard D,L-Dab mixture **(D)** P2 hydrolysate. The ratio of L-Dab to D-Dab was approximately 5:1 in P2. This suggests that there is one D-Dab in the compound.

**FIGURE 4:**
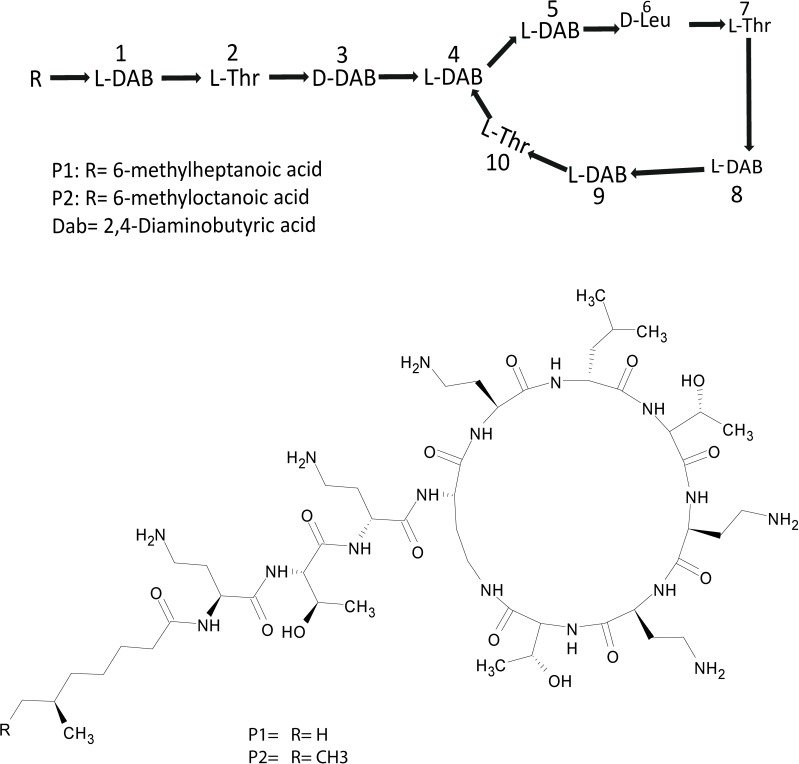
Proposed 2D structure of P1 and P2.

Extracts derived from more than hundred isolates were screened for antimicrobial activity using agar well diffusion assay. The strain PV3-16, isolated from river sediment, showed antagonistic activity only against Gram-negative bacteria. Later on, it was identified as *Paenibacillus dendritiformis* species (99.98% similarity) based on 16S rRNA gene sequencing (GenBank accession number: MH472941). The strain PV3-16 formed a tip-splitting pattern (Supplementary Figure S2) during the colony development on 1.5% agar medium containing 0.2% peptone as described previously in the first report of this species description (Tcherpakov et al., 1999). The pairwise sequence of 16S rRNA gene extracted from whole genome of this strain showed 100% similarity with *P. dendritiformis* C454. Then, we calculated the Average Nucleotide Identity (ANI) of our strain with the type strain. It was found to be 97.80%. Moreover, we performed digital DNA-DNA hybridization (dDDH) of these strains and the value obtained was 83.9% which is above the gold standard threshold value of 70% for the delineation of species (Auch et al., 2010). Two compounds initially named P1 and P2, were isolated and purified from this strain through a series of chromatographic techniques (Figure 1). The compounds, when subjected to mass spectroscopic analysis, showed m/z values of 1143.69 [M+H]^+^ and 1157.69 [M+H]^+^ respectively, differing in the mass of 14Da (Figure 1). Both compounds showed a similar fragmentation in tandem mass spectroscopy suggesting that these compounds might be two different variants of the same molecule. We searched for the compounds having similar molecular masses in literature and speculated that these compounds might belong to polymyxin class. Also, *Paenibacillus* genus is well reported for the production of polymyxins (Martin et al., 2003; DeCrescenzo Henriksen et al., 2007; Shaheen et al., 2011; Niu et al., 2013). We used polymyxin B and E as control and compared their MS and MS/MS spectra with our compounds (data not shown). The amino acid analysis also supported the MS/MS data (Figure 2) and the amino acid composition matched with polymyxin M (Martin et al., 2003). As in other polymyxins, there are variants reported which differ in N-terminal fatty acid chain length. The major components of polymyxin B and E are B_1_ and B_2_, and E_1_ and E_2_ respectively. B_1_ and E_1_ contain 6-methyloctanoic acid while other two contain isooctanoic acid (i.e., 6-methylheptanoic acid) making a difference of 14 Da (Storm et al., 1977; Cui et al., 2018). We believed that our compounds P1 and P2 were also two different variants of the same molecule differing at their fatty acid moiety. But for polymyxin M, only 1157 Da mass was reported by Martin et al.; no information on the mass of 1143 Da was described (Martin et al., 2003). To solve this paradox, we acquired different one- and two-dimensional NMR spectra (Supplementary Figures S3, S4) of both compounds and found that both compounds differed only in their fatty acid moiety. NMR spectra were consistent with polymyxin M or mattacin (Martin et al., 2003). Upon further literature survey, we came across polymyxin A which has the same mass as polymyxin M but differing with stereochemistry of one amino acid, i.e., Dab residue at the 3rd position, polymyxin A has D-Dab while polymyxin M contains L-Dab (Choi et al., 2009; Shaheen et al., 2011; Cui et al., 2018). To differentiate between polymyxin M and A, we subjected our strain to whole genome sequencing (deposited under GenBank accession number QKVW00000000) and the draft genome was uploaded on AntiSMASH to locate the secondary metabolite clusters. We found an NRPS cluster of polymyxin which was dispersed among seven contigs (Supplementary Figure S5A). Upon pairwise alignment of gene clusters, we concluded that our cluster showed maximum similarity with the polymyxin A gene cluster from the strain *Paenibacillus polymyxa* E681 (Choi et al., 2009). There was the presence of an epimerase gene next to the adenylation domain of 3rd Dab residue (Supplementary Figure S5B). This enzyme may be responsible for the conversion of L-Dab to D-Dab. We confirmed the stereochemistry of this residue using Marfey’s analysis (Figure 3). Compounds P1 and P2 contained one D isomer and five L isomers of Dab residue consistent with polymyxin A_2_ and A_1_ respectively. Figure 4 shows the proposed 2D structure of both the compounds.

### Determination of MIC

Table 1 shows the MICs of P1 and P2 compounds along with reference polymyxin B and E against a wide panel of quality control strains. All compounds showed excellent activity against most of the Gram-negative strains except *Vibrio* sp. For quality control strains, there was no significant difference between MIC values of these compounds and reference polymyxin B and E. We also assayed a battery of 31 clinical MDR isolates, which included four ESKAPE pathogens viz. *E. coli*, *K. pneumoniae*, *A. baumanii*, and *P. aeruginosa*, for their susceptibility toward P1 and P2 and compared the results with those obtained for standard polymyxins. In six of the strains, MIC values for all the compounds was same but the compound P1 was generally the more potent of four polymyxin used as it showed 2–4 fold less MIC in 25 clinical isolates (Table 2). The antibiotic-susceptibility of clinical strains is provided in Supplementary Table S1.

**Table 1:** MIC data of P1 and P2 against quality control strains.

Sr. No	Strain name	Strain designation	MIC (μg/mL)			
			P1	P2	PB	PE
1	*Acinetobacter baumannii*	ATCC 19606	0.5	1	1	1
2	*Escherichia coli*	ATCC 25922	1	1	1	1
3	*Escherichia coli*	ATCC 35218	0.25	0.5	0.5	0.5
4	*Klebsiella pneumoniae*	ATCC BAA1706	0.5	1	1	0.5
5	*Klebsiella pneumoniae*	ATCC 700603	1	1	1	1
6	*Klebsiella pneumoniae*	ATCC 29665	0.5	1	1	0.5
7	*Klebsiella pneumoniae*	ATCC- BAA 2146	0.5	1	1	0.5
8	*Pseudomonas aeruginosa*	ATCC 27853	0.5	1	1	1
9	*Vibrio parahaemolyticus*	ATCC17802	1	1	1	1
10	*Vibrio cholerae*	MTCC 3906	>16	8	8	>16
11	*Bacillus subtilis*	ATCC 6633	16	8	4	16
12	*Staphylococcus aureus*	ATCC 25923	>16	>16	16	>16
13	*Enterococcus faecalis*	ATCC 51299	>16	>16	>16	>16

*PB, Polymyxin B; PE, Polymyxin*.

**Table 2:** MIC of isolated compounds in MDR clinical isolates.

Sr. No.	Strain	MIC (μg/mL)				Sr. No.	Strain	MIC (μg/mL)			
	*K. Pneumoniae*	P1	P2	PB	PE		*E. coli*	P1	P2	PB	PE
1	GMCH 16	0.5	0.5	0.5	1	18	7534	**0.25**	0.5	1	0.5
2	GMCH10	1	1	1	1	19	9062	**0.25**	1	1	1
3	1573	**0.5**	0.5	1	1	20	7210	**0.25**	1	1	1
4	GMCH13	**0.5**	0.5	1	1	21	14363	**0.25**	0.5	0.5	0.5
5	GMCH12	**0.5**	1	1	1	22	7932	**0.25**	1	1	1
6	1428	**1**	2	2	2	23	3185	**0.25**	0.5	0.5	0.5
7	GMCH04	**0.25**	0.5	0.5	0.5	24	14504	**0.25**	0.5	1	1
8	GMCH11	1	1	1	0.5	25	13425	**0.25**	1	1	1
9	827	**0.5**	1	1	1	26	14084	**0.25**	0.5	0.5	0.5
10	B8	**0.5**	1	1	1							

	***P. aeruginosa***						***A. baumannii***				

11	GMCH06	**0.5**	1	1	1	27	GMCH 14	**0.5**	1	1	1
12	PA1	**1**	2	2	2	28	GMCH18	**0.25**	0.5	1	1
13	PA2	**1**	2	2	2	29	GMCH05	1	1	1	1
14	PA3	**1**	2	2	2	30	AB1	**0.5**	1	1	1
15	PA4	1	1	1	1	31	AB2	1	1	1	1
16	PA5	**1**	2	2	2						
17	PA6	**1**	2	2	2						

*PB, Polymyxin B; PE, Polymyxin. The bold values highlights the lower MIC of the compound P1 than the standard polymyxins*.

### Time-Kill Kinetics of P1 and P2

**FIGURE 5:**
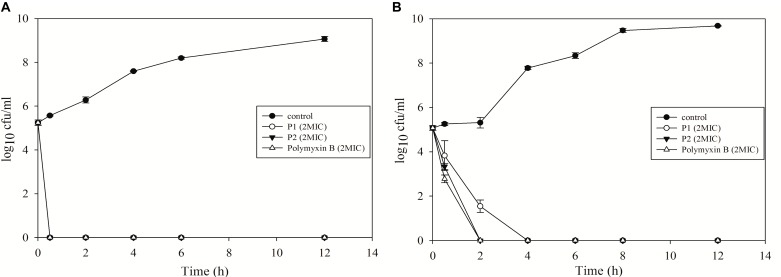
Time-kill kinetics of P1 and P2 against **(A)** *E. coli* ATCC 25922 and **(B)** *P. aeruginosa* ATCC 27853. The experiment was carried out in triplicate and two biological repeats were performed. Data plotted as mean ± SD.

Both compounds showed excellent bactericidal activity against *E. coli* and *P. aeruginosa* and killing time was equivalent to polymyxin B (Figure 5). *E. coli* was completely killed in 30 min in presence of 2xMIC for all the compounds. While P2 and polymyxin B killed *P. aeruginosa* in 2 h, P1 took 4 h to kill. P1 acted slowly on *P. aeruginosa* but MIC value was twofold less for P1. This data suggests that killing kinetics not just depends on MIC value, it varies from species to species and depends on the membrane structure of different bacteria.

### Membrane Permeabilization Assays

**FIGURE 6:**
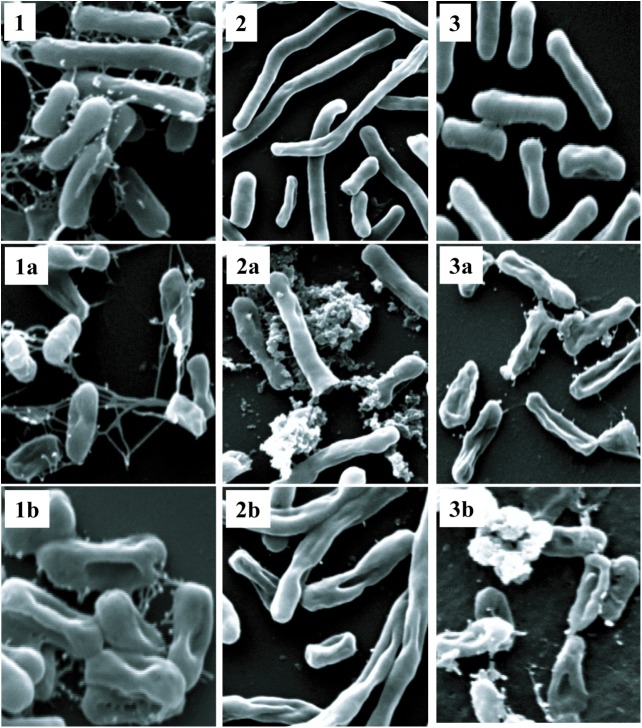
Scanning electron microscopy. Upper panel shows control cells without treatment **(1)** *K. pneumoniae* BAA-1706, **(2)** *A. baumannii* ATCC 19606 and **(3)** *P. aeruginosa* ATCC 27853. P1 and P2 were used at a concentration of 10 μg/ml at 37°C. The optimized treatment time was 45 min for *K. pneumoniae* and *A. baumanii* while *P. aeruginosa* was incubated for 2 h. **(1a,2a,3a)** are cells treated with P1, and **(1b,2b,3b)** show the cells treated with P2.

**FIGURE 7:**
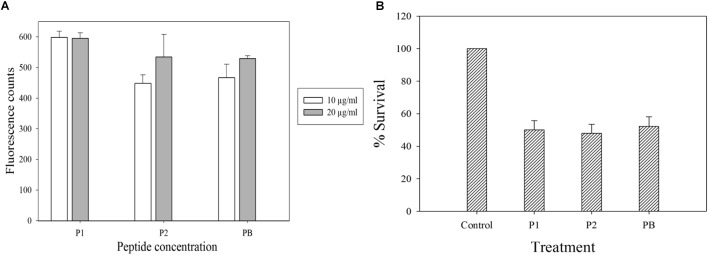
Membrane permeabilization assays with *E. coli* ATCC 25922. **(A)** NPN uptake assay to check the outer membrane permeabilization. **(B)** Inner membrane permeabilization using fluorescent dyes SYTO9 and PI; the concentration of each compound is 10 μg/ml. Control in **(B)** is cells without peptide treatment. Data is plotted as Mean ± SD of three replicates. Two biological repeats were performed.

Polymyxins bind to the LPS component of cell membrane and kill the bacteria by creating pores in the membrane (Newton, 1956). Initially, we performed electron microscopy to see the effect of antimicrobial compounds on bacterial membrane. *K. pneumoniae* BAA-1706, *A. baumannii* ATCC 19606 and *P. aeruginosa* ATCC 27853 were chosen for this experiment. Scanning electron microscopy images (Figure 6) clearly show the membrane damage in Gram-negative bacteria. Some cells were completely ruptured in presence of antimicrobial compounds. To check the permeabilization of the outer membrane, we measured NPN uptake in terms of fluorescence. As seen in Figure 7A, there was no significant difference in fluorescence intensity of both compounds when compared with polymyxin B which is considered as strong outer membrane permeabilizing agent. P1 showed slightly higher NPN uptake. Also, inner membrane permeability results show that both compounds have similar potential to penetrate in the membrane of bacteria and kill them (Figure 7B). The results were comparable to those obtained with polymyxin B. In 1 h, all compounds were able to permeabilize 50% of the cells.

### Isothermal Titration Calorimetry (ITC)

**FIGURE 8:**
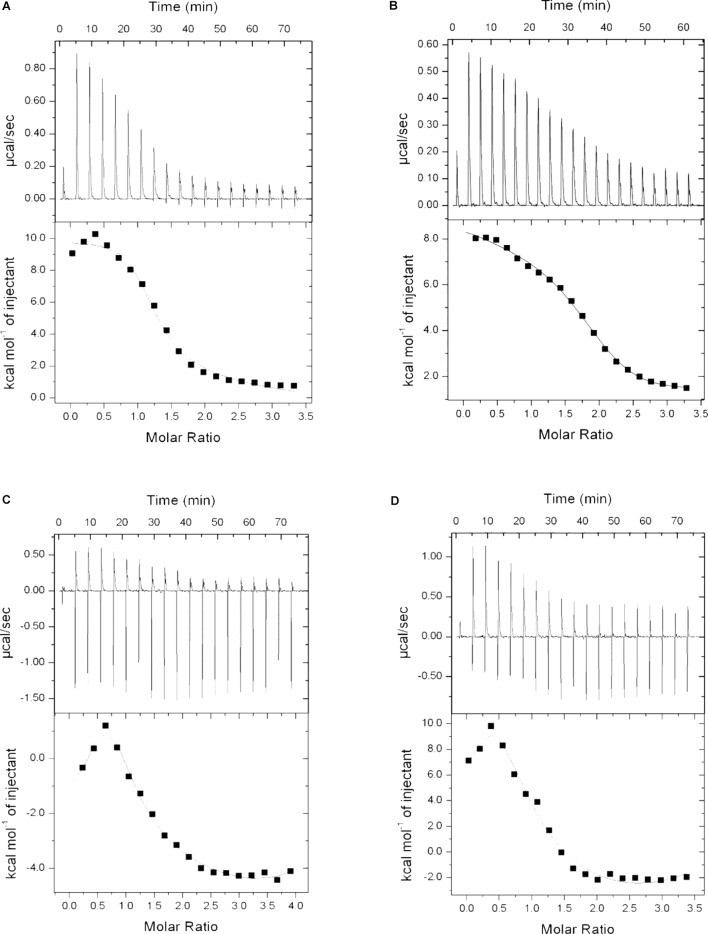
Binding isotherms of P1 **(A)**, P2 **(B)**, polymyxin B **(C)**, and polymyxin E **(D)** with lipopolysaccharide. LPS concentration was 0.05 mM where all four compounds were used at 1 mg/ml (0.83–0.87 mM). Dissociation constant (Kd) determined as: P1 = 9.52E-07, P2 = 2.94E-06, Polymyxin B = 3.39E-06 and Polymyxin E = 7.04E-06.

To examine if P1 and P2 act in a similar way as described for standard polymyxins, isothermal calorimetry was performed. LPS was titrated with P1, P2, polymyxin B and E, and binding isotherms were obtained. It is evident from the Figure 8 that P1 and P2 bind to the LPS in a similar pattern as polymyxin B and E but binding affinities were different for all compounds tested. As mentioned in the figure legend, the Kd value for P1 is smallest among all compounds which means that P1 has higher affinity toward LPS. This also corroborates with the lower MIC of P1 in *E. coli* strains. Our results resemble the binding isotherms published by Martin et al. (2003) for polymyxin B and polymyxin M.

### Mammalian Cytotoxicity

**FIGURE 9:**
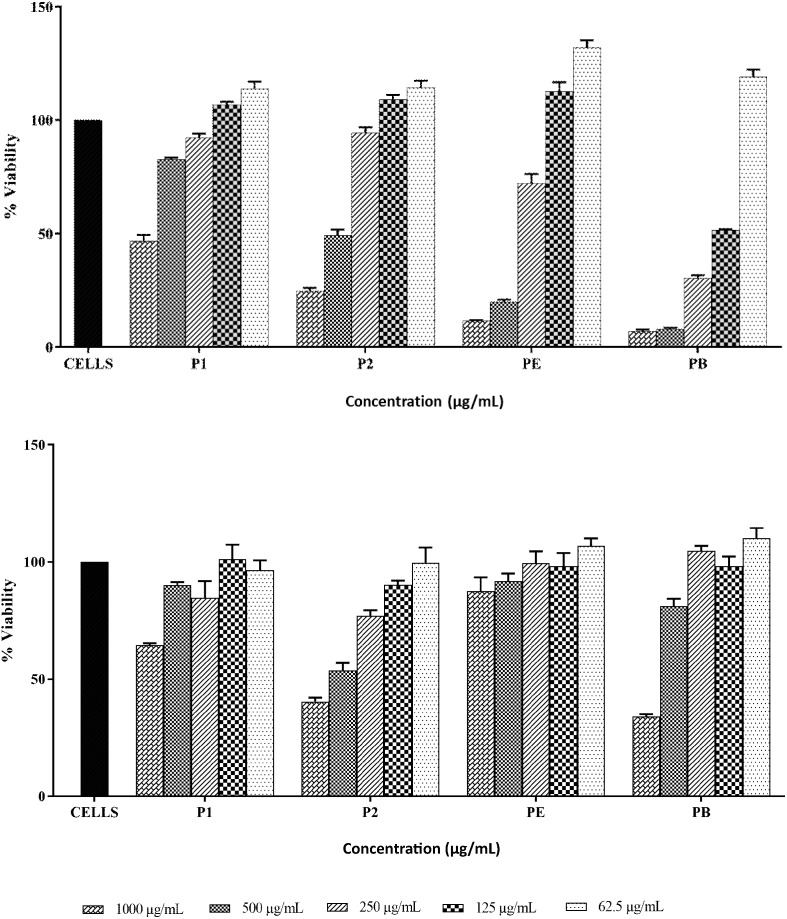
Cytotoxicity of P1and P2 at various concentrations against THP-1 cell line (top) and HEK293 cell line (bottom). PE, polymyxin E and PB, polymyxin B. The experiment was performed in triplicate and is representative of two biological repeats. Data plotted as Mean ± SD.

THP-1 and HEK293 cell lines were used to assess the toxicity of both compounds (Figures 9A,B). P1 exhibited IC_50_ value at approximately 1000 μg/ml against THP-1 cells while IC_50_ values for P2, polymyxin B and polymyxin E were 500 μg/ml, 125 μg/ml, and >250 μg/ml respectively. HEK293 cells were more resistant to polymyxins. P1 and polymyxin E displayed similar toxicity with IC_50_ at above 1000 μg/ml. P2 showed the highest toxicity among four compounds against HEK cells.

## Discussion

Polymyxins are an essential group of antibiotics used as the last therapeutic option to treat infections caused by carbapenem-resistant *Enterobacteriaceae*, MDR *A. baumannii*, and *P. aeruginosa*. Due to significant nephrotoxicity, they fell out of favor in the 1970s but reappeared in early 2000s when no other drugs were available to cure severe Gram-negative infections (Landman et al., 2008; Pogue et al., 2017). Out of all polymyxins discovered to date, only polymyxins B and E are used in clinical practice. The plausible explanation for this is that no other polymyxin molecule has been studied in sufficient detail to show its clinical relevance (Velkov et al., 2013).

In this study, we have isolated a bacterial strain PV3-16 from soil sediment which was identified as *Paenibacillus dendritiformis* based on 16S rRNA gene sequencing and whole genome analysis. This strain was showing antagonistic activity against Gram-negative pathogens. Since there was no report of purification of antimicrobial compound from this species previously, we decided to work on this strain. We isolated and characterized two antimicrobial compounds, P1 and P2 from this strain using various chromatographic and analytical techniques. Since the compounds did not differ much with respect to their charge and molecular weights as explained next, it was quite difficult to separate them in HPLC. We partially purified the antimicrobial compounds from rest of the junk or impurities using cation-exchange chromatography. Afterward, the reversed-phase HPLC with very gentle slope of gradient and a stretch of 20–35% B over a long time period successfully separated the two compounds (Supplementary Figure S1) Mass spectroscopy, amino acid analysis, and NMR spectroscopy revealed that both compounds were similar in their fragmentation pattern and amino acid composition and suggested that they belong to polymyxin family. The spectroscopic data were consistent with polymyxin M (Martin et al., 2003). Further, we performed the whole genome sequencing of our strain, PV3-16 and searched for polymyxin gene cluster. Upon pairwise alignment, the cluster showed maximum homology with polymyxin A gene cluster published by Choi et al. (2009). Polymyxin A has same molecular weight and amino acid composition as polymyxin M (Shaheen et al., 2011) but differs with stereochemistry of Dab residue at 3rd position. Considering the biosynthetic gene cluster and the stereochemical analysis of our compound using Marfey’s reagent, we propose that these two compounds, named P1 and P2 are polymyxin A_2_ and A_1_ respectively. *In vitro* efficacy studies of these compounds proved that both molecules are as efficacious as polymyxins B and E. Moreover, P1 showed two to four folds less MIC against most of the clinical pathogens tested. We also checked the binding isotherms of both compounds with LPS, a major component present in the outer membrane of Gram-negative bacteria, and found that P1 possessed higher affinity toward LPS. This data corroborates with the lower MIC value obtained for P1. To study the *in vitro* cytotoxicity of these compounds, we selected THP-1 cell line and HEK293 cell line. We deduced that P1 is least toxic to immune cells (THP-1 cell line) while in kidney cell line, P1 showed similar results as polymyxin E. In the previous report published by Roberts et al. (2015) the authors studied the *in vitro* toxicity of major components of polymyxin B and E in HK2 (human proximal kidney cells) and found the *in vitro* toxicity pattern as E_2_ < E_1_ < B_2_ < B_1_. The possible reason for this behavior could be the lowest hydrophobicity of E_2_. As the hydrophobicity of polymyxin increases, the toxicity also goes higher. This explanation also fits well for our compounds since P1 is less hydrophobic than polymyxin E_2_ (P1 has one extra threonine in place of leucine in polymyxin E_2_, making P1 more polar than E_2_). Also, in a recent paper published by one group in China (Cui et al., 2018), authors studied the efficacy and toxicity of different polymyxin molecules and they also observed that polymyxin A was equally efficacious as compared to polymyxin B and E. Polymyxin A showed less toxicity than all polymyxins studied except polymyxin S_2_ and D_2_ against Vero kidney cell line. Moreover, the main focus of polymyxin toxicity has been its nephrotoxicity or neurotoxicity in that report and previous literature (Falagas and Kasiakou, 2006; Landman et al., 2008; Justo and Bosso, 2015; Pogue et al., 2017). Surprisingly we found that THP-1 cells were more sensitive to polymyxins than kidney cells. So, the dose regimen designed based on the nephrotoxicity data may have a substantial effect on immune cells. But further experiments need to be performed to clearly see the effect of such compounds on immune system. In a clinical data published by one Indian hospital (Ghafur et al., 2014), there was complete microbiological clearance upon the higher dose of colistin (i.e., polymyxin E) combination- or mono-therapy in patients infected with bacteremic or non-bacteremic Pan-drug resistant Gram-negative bacteria but still, after few months, five out of eight patients died. This may be in part due to the significant damage of immune system at a high dose of polymyxin E and other antibiotics used in combination. So, we should be extra cautious while designing the dose-regimen and we need to evaluate the overall toxicity of polymyxins other than its reported nephrotoxicity and neurotoxicity.

To conclude, we purified the two major components of polymyxin A and studied them *in vitro*. Polymyxin A seems to be equally efficacious and less toxic than polymyxin B and colistin based on these *in vitro* studies, especially against immune cells. Whether polymyxin A will work similarly under *in vivo* conditions and will have clinical potential needs to be investigated further in details. Also, pharmacokinetic and pharmacodynamic properties should be studied to develop it as a drug candidate, an alternative to standard polymyxins.

## Ethics Statement

The Authors have not performed any study on human subjects or animal models.

## Author Contributions

MJ contributed to the isolation and screening of microbes. MJ, HKR, AS, MK, NM, PPP, and PJ performed the experiments. MJ and MK wrote and edited the manuscript. AA performed the NMR studies. PBP, MR, AA, and HN designed the study plan, executed the plan, and analyzed the data.

## Conflict of Interest Statement

The authors declare that the research was conducted in the absence of any commercial or financial relationships that could be construed as a potential conflict of interest.
