# Supplementary material for: Purification, Characterization and in vitro Evaluation of Polymyxin A From Paenibacillus dendritiformis: An Underexplored Member of the Polymyxin Family
Source: Front Microbiol. 2018 Nov 23;9:2864. doi: 10.3389/fmicb.2018.02864 (PMC6265310; doi:10.3389/fmicb.2018.02864)
Supplement: Supplementary file 1 [file Data_Sheet_1.pdf]

Purification, characterization and *in vitro* evaluation of polymyxin A from *Paenibacillus dendritiformis*: An underexplored member of the polymyxin family

Manoj Jangra[1], Harmandeep Kaur Randhawa[1], Manpreet Kaur[1], Anugya Srivastava[1], Navdezda Maurya[1], Prashant P Patil[2], Pallavi Jaswal[3], Ashish Arora[4], Prabhu B Patil[2], Manoj Raje[3], Hemraj Nandanwar[1*]

[1]Clinical Microbiology & Bioactive Screening Laboratory, CSIR-Institute of Microbial Technology, Sector -39A, Chandigarh, India

[2] Bacterial Genetics & Genomics Laboratory, CSIR-Institute of Microbial Technology, Sector -39A, Chandigarh, India

[3]Cell Biology and Microscopy Laboratory, CSIR-Institute of Microbial Technology, Sector -39A, Chandigarh, India

[4] Molecular and Structural Biology Division, CSIR-Central Drug Research Institute, Mahatma Gandhi Marg, Lucknow, India

***Address for correspondence**

Dr. Hemraj Nandanwar

CSIR - Institute of Microbial Technology, Sector -39A, Chandigarh, India, 160036

E-mail: hemraj@imtech.res.in

Telephone: +91-172-6665338 Fax: +91-172-2690585/2690632

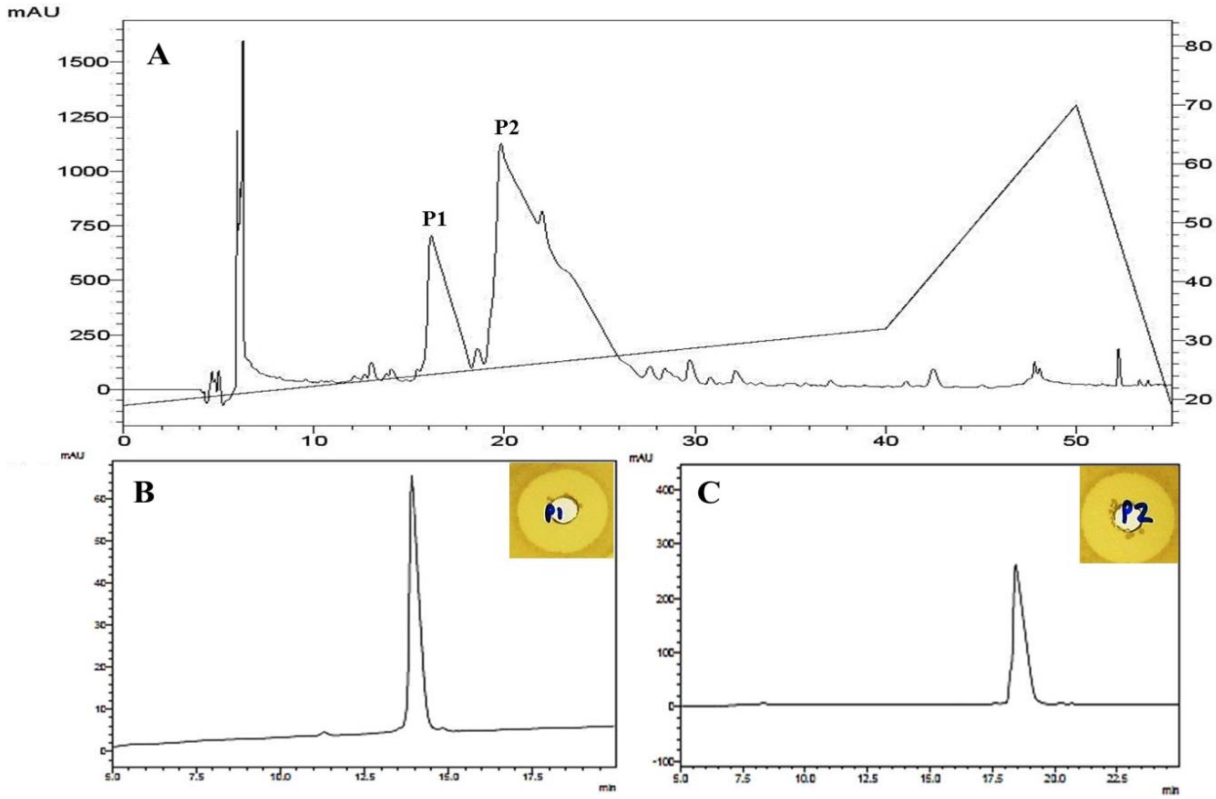

Supplementary Figure S1 (A) HPLC profile of cation dialysate. (B) Peak 1 and (C) Peak 2 purified. Insets show the antimicrobial activity of both compounds

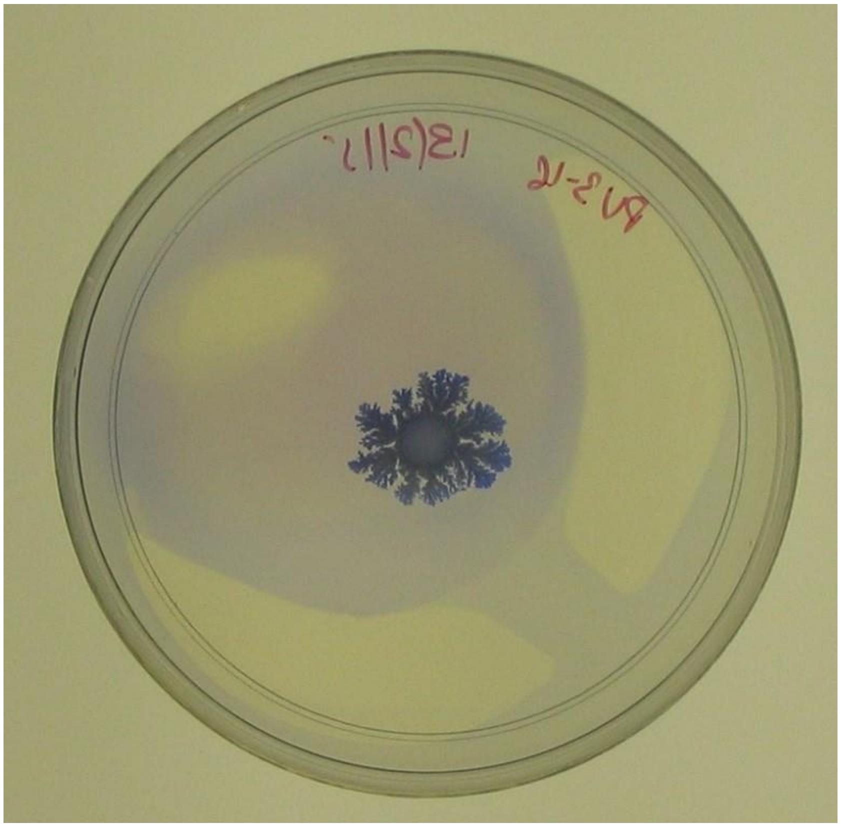

Supplementary Figure S2 Appearance of colony of PV3-16 strain in tip-splitting pattern on 1.5% agar medium containing 0.2% peptone. 5µl log-phase culture was spotted and incubated at 37 °C. The plate shown here is after 3-days incubation and then stained with crystal violet dye to visualize the patterns

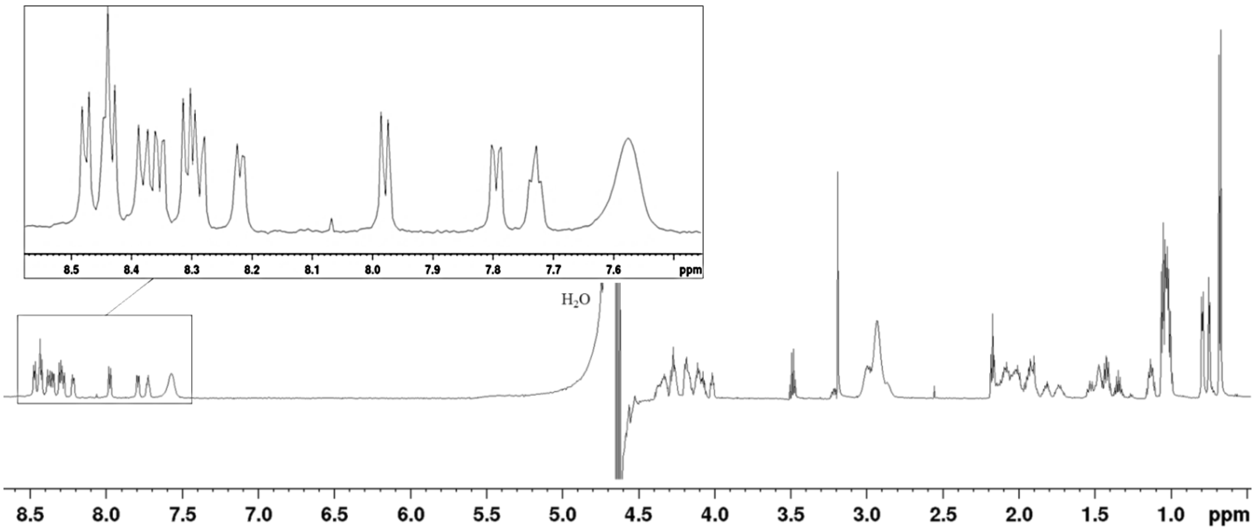

(A)

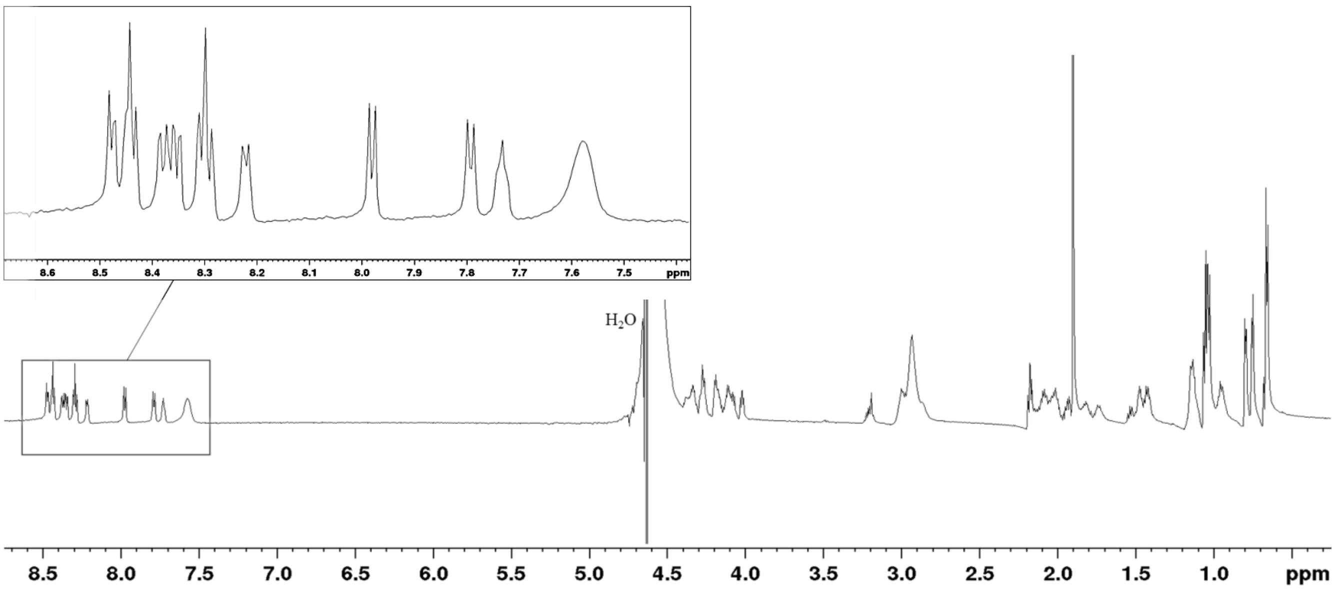

(B)

Supplementary Figure S3 One dimensional 1H NMR spectra of peak1 (A) and peak 2 (B) with the expansion of amide region

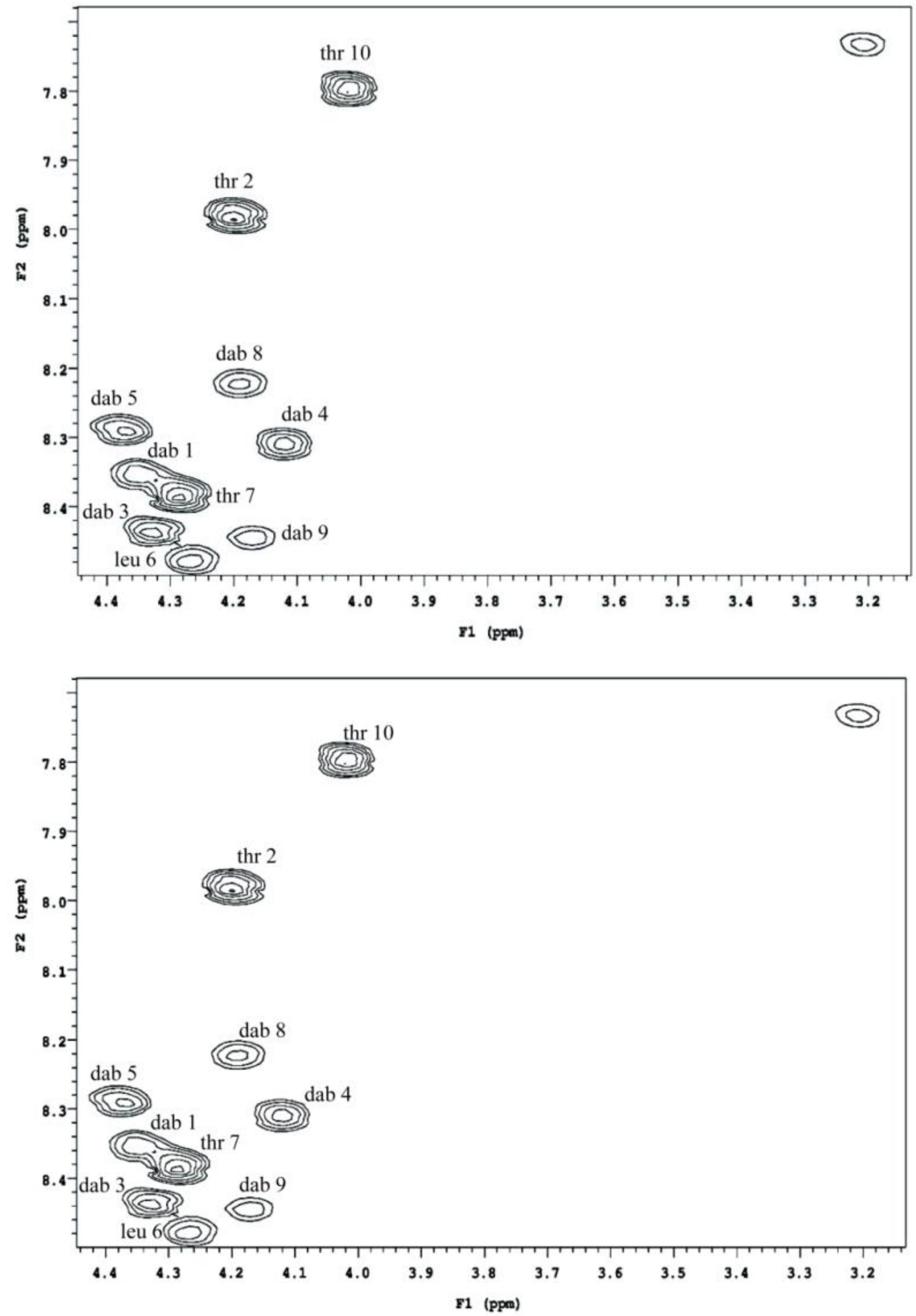

Supplementary Figure S4 HH-COSY showing the α-H/amide-H correlations. Upper panel (P 1) and lower panel (P 2)

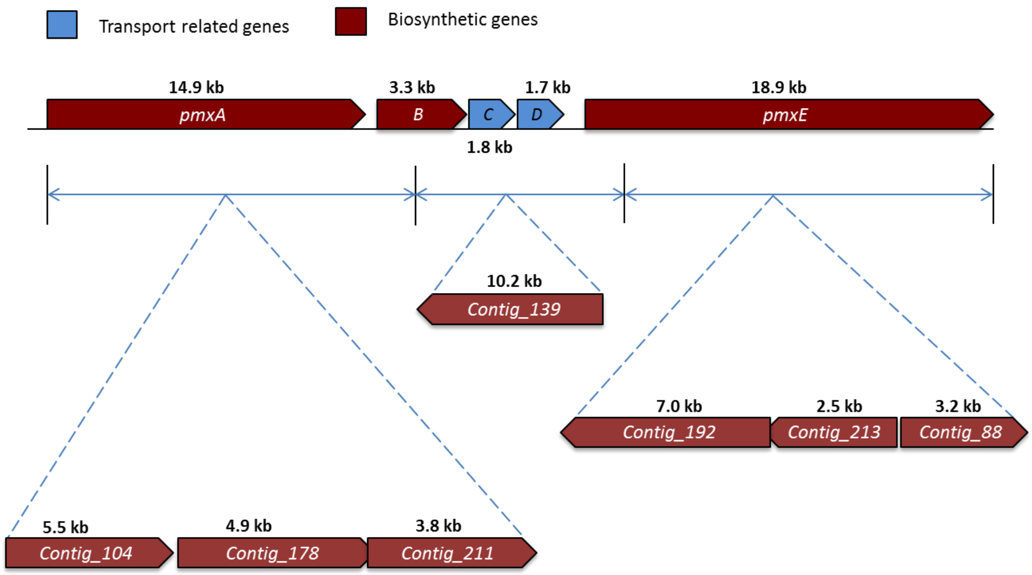

(a)

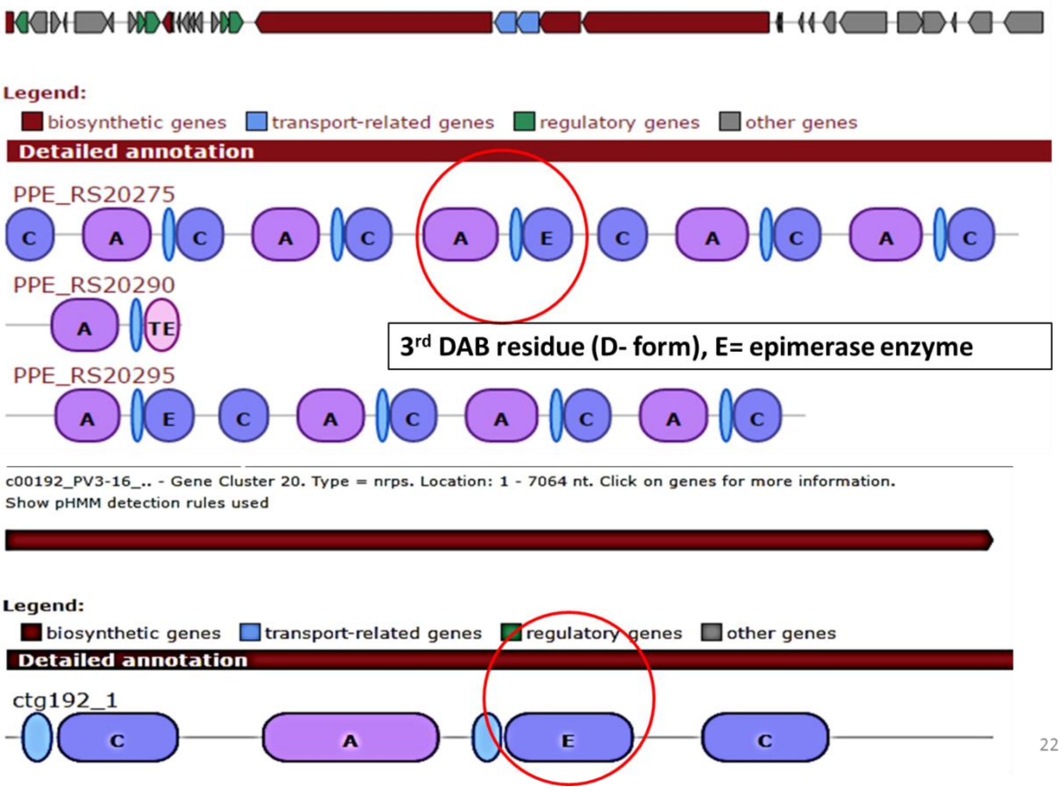

(b)

Supplementary Figure S5 Biosynthetic gene cluster for polymyxin A. (a) Upper panel shows the polymyxin gene cluster reported in the literature by Choi et al. Gene cluster in PV3-16 strain is dispersed in seven contigs. (b) Detailed annotation of PmxE gene showing gene for epimerase enzyme responsible for incorporation of D-Dab at third position. 4

Supplementary Table-S1 Antibiotic-susceptibility pattern of clinical isolates

**Clinical isolate**	**CAZ 30**	**TE 30**	**AK 30**	**PI 100**	**S 10**	**FO 200**	**NIT 300**	**CTX 30**	**CIP 5**	**NX 10**	**CXM 30**	**TR 5**	**CFM 5**	**AMC 30**	**PIT 100/10**	**A/S 10/10**
3185	0<br>R	10<br>R	21<br>S	0<br>R	20<br>S	34<br>S	22<br>S	0<br>R	0<br>R	10<br>R	0<br>R	0<br>R	0<br>R	10<br>R	18<br>I	12<br>I
7932	13<br>R	9<br>R	20<br>S	9<br>R	22<br>S	31<br>S	19<br>S	10<br>R	8<br>R	9<br>R	0<br>R	0<br>R	0<br>R	10<br>R	26<br>S	14<br>I
14504	17<br>R	0<br>R	20<br>S	13<br>R	11<br>R	31<br>S	20<br>S	9<br>R	0<br>R	11<br>R	0<br>R	0<br>R	0<br>R	12<br>R	27<br>S	14<br>I
14363	0<br>R	10<br>R	27<br>S	7<br>R	15<br>S	34<br>S	23<br>S	0<br>R	13<br>R	11<br>R	0<br>R	0<br>R	0<br>R	10<br>R	24<br>S	13<br>I
9062	9<br>R	15<br>R	19<br>S	0<br>R	18<br>S	34<br>S	23<br>S	0<br>R	0<br>R	9<br>R	0<br>R	0<br>R	0<br>R	0<br>R	20<br>I	11<br>R
7210	16<br>R	0<br>R	21<br>S	12<br>R	18<br>R	36<br>S	22<br>S	11<br>R	0<br>R	5<br>R	0<br>R	0<br>R	0<br>R	12<br>R	27<br>S	16<br>S
7534	16<br>R	16<br>R	21<br>S	16<br>R	11<br>R	31<br>S	16<br>I	15<br>R	0<br>R	12<br>R	0<br>R	0<br>R	0<br>R	12<br>R	25<br>S	17<br>S
13425	0<br>R	15<br>R	27<br>S	8<br>R	0<br>R	37<br>S	23<br>S	10<br>R	13<br>R	11<br>R	0<br>R	31<br>S	21<br>S	11<br>R	23<br>S	13<br>I
14084	13<br>R	13<br>R	26<br>S	11<br>R	22<br>R	40<br>S	28<br>S	15<br>R	12<br>R	10<br>R	0<br>R	0<br>R	0<br>R	17<br>I	31<br>S	17<br>S
GMCH16	0<br>R	11<br>R	0<br>R	0<br>R	22<br>S	23<br>S	8<br>R	8<br>R	10<br>R	0<br>R	0<br>R	0<br>R	0<br>R	0<br>R	12<br>R	0<br>R
GMCH10	0<br>R	11<br>R	9<br>R	0<br>R	0<br>R	18<br>S	10<br>R	0<br>R	14<br>R	17<br>S	0<br>R	0<br>R	0<br>R	0<br>R	13<br>R	0<br>R
1573	0<br>R	22<br>S	0<br>R	0<br>R	19<br>S	24<br>S	11<br>R	0<br>R	9<br>R	0<br>R	0<br>R	0<br>R	0<br>R	0<br>R	0<br>R	0<br>R
GMCH13	13<br>R	10<br>R	22<br>S	9<br>R	9<br>R	10<br>R	11<br>R	13<br>R	10<br>R	10<br>R	0<br>R	0<br>R	0<br>R	9<br>R	17<br>R	13<br>I
GMCH12	0<br>R	0<br>R	0<br>R	0<br>R	22<br>S	23<br>S	9<br>R	0<br>R	11<br>R	12<br>R	0<br>R	0<br>R	0<br>R	0<br>R	0<br>R	0<br>R
1428	0<br>R	24<br>S	0<br>R	9<br>R	0<br>R	26<br>S	14<br>R	0<br>R	12<br>R	13<br>I	0<br>R	0<br>R	0<br>R	0<br>R	13<br>R	0<br>R
GMCH04	0<br>R	17<br>S	0<br>R	0<br>R	12<br>I	16<br>S	0<br>R	0<br>R	0<br>R	0<br>R	0<br>R	0<br>R	0<br>R	0<br>R	0<br>R	0<br>R
GMCH11	0<br>R	22<br>S	0<br>R	0<br>R	12<br>I	18<br>S	11<br>R	0<br>R	0<br>R	0<br>R	0<br>R	28<br>S	0<br>R	0<br>R	10<br>R	0<br>R
827	0<br>R	19<br>S	0<br>R	0<br>R	20<br>S	22<br>S	0<br>R	0<br>R	15<br>R	13<br>I	0<br>R	27<br>S	0<br>R	0<br>R	0<br>R	0<br>R
B8	0<br>R	25<br>S	0<br>R	0<br>R	12<br>I	23<br>S	13<br>R	0<br>R	11<br>R	12<br>R	0<br>R	0<br>R	0<br>R	0<br>R	0<br>R	0<br>R
GMCH18	0<br>R	36<br>S	13<br>R	0<br>R	0<br>R	24<br>S	0<br>R	0<br>R	0<br>R	12<br>R	0<br>R	18<br>S	0<br>R	10<br>R	13<br>R	18<br>S
GMCH05	9<br>R	0<br>R	13<br>R	0<br>R	0<br>R	19<br>S	0<br>R	0<br>R	14<br>R	17<br>S	0<br>R	0<br>R	0<br>R	0<br>R	12<br>R	15<br>S
GMCH 14	9<br>R	0<br>R	12<br>R	0<br>R	0<br>R	25<br>S	0<br>R	0<br>R	15<br>R	21<br>S	0<br>R	0<br>R	0<br>R	14<br>I	17<br>R	22<br>S
GMCH06	0<br>R	11<br>R	0<br>R	10<br>R	26<br>S	29<br>S	0<br>R	16<br>R	0<br>R	0<br>R	0<br>R	0<br>R	0<br>R	10<br>R	17<br>R	0<br>R

Clinical isolate	CAZ 30	TE 30	AK 30	PI 100	S 10	FO 200	NIT 300	CTX 30	CIP 5	NX 10	CXM 30	TR 5	CFM 5	AMC 30	PIT 100/10	A/S 10/10
PA1	0 R	ND	10 R	16 R	ND	ND	0 R	11 R	0 R	11 R	0 R	ND	ND	ND	17 R	ND
PA2	0 R	ND	10 S	16 R	ND	ND	0 R	22 S	0 R	10 R	0 R	ND	ND	ND	17 R	ND
PA3	31 S	ND	22 S	32 S	ND	ND	10 R	29 S	19 R	28 S	21 R	ND	ND	ND	36 S	ND
PA4	31 S	ND	13 R	28 S	ND	ND	0 R	26 S	11 R	0 R	20 S	ND	ND	ND	27 S	ND
PA5	0 R	ND	0 R	16 R	ND	ND	0 R	12 R	10 R	10 R	0 R	ND	ND	ND	17 R	ND
PA6	32 S	ND	33 S	30 S	ND	ND	0 R	30 S	20 S	32 S	24 S	ND	ND	ND	31 S	ND

The results were observed as zone of inhibition around the disc as denoted by the value in the boxes. R= Resistant; S= Sensitive; I= Intermediate; ND=not determined

**CAZ 30 µg**	Ceftazidime
**TE 30 µg**	Tetracyline
**AK 30 µg**	Amikacin
**PI 100 µg**	Piperacillin
**S 10 µg**	Streptomycin
**FO 200 µg**	Fosfomycin
**NIT 300 µg**	Nitrofurantoin
**CTX 30 µg**	Cefotaxime
**CIP 5 µg**	Ciprofloxacin
**NX 10 µg**	Norfloxacin
**CXM 30 µg**	Cefuroxime
**TR 5 µg**	Trimethoprim
**CFM 5 µg**	Cefixime
**AMC 30 µg**	Amoxyclav
**PIT 100/10 µg**	Piperacillin/Tazobactam
**A/S 10/10 µg**	Ampicillin/Sulbactam
